# SARS-CoV-2 infection causes immunodeficiency in recovered patients by downregulating CD19 expression in B cells via enhancing B-cell metabolism

**DOI:** 10.1038/s41392-021-00749-3

**Published:** 2021-09-22

**Authors:** Yukai Jing, Li Luo, Ying Chen, Lisa S. Westerberg, Peng Zhou, Zhiping Xu, Andrés A. Herrada, Chan-Sik Park, Masato Kubo, Heng Mei, Yu Hu, Pamela Pui-Wah Lee, Bing Zheng, Zhiwei Sui, Wei Xiao, Quan Gong, Zhongxin Lu, Chaohong Liu

**Affiliations:** 1 Department of Medical Laboratory, The Central Hospital of Wuhan, Tongji Medical College, Huazhong University of Science and Technology, Wuhan, People’s Republic of China; 2 Department of Emergency, Shanxi Bethune Hospital, Shanxi Academy of Medical Sciences, Tongji Shanxi Hospital, Third Hospital of Shanxi Medical University, Taiyuan, People’s Republic of China; 3 Tongji Hospital, Tongji Medical College, Huazhong University of Science and Technology, Wuhan, People’s Republic of China; 4 Department of Pathogen Biology, School of Basic Medicine, Tongji Medical College, Huazhong University of Science and Technology, Wuhan, People’s Republic of China; 5 Center for Biosafety Mega-Science, Wuhan Institute of Virology, Chinese Academy of Sciences, Wuhan, People’s Republic of China; 6 Department of Microbiology Tumor and Cell Biology, Karolinska Institute, Stockholm, Sweden; 7 Wuhan Metware Biotechnology Co., Ltd, Wuhan, People’s Republic of China; 8 Lymphatic and Inflammation Research Laboratory, Facultad de Ciencias de la Salud, Instituto de Ciencias Biomédicas, Universidad Autónoma de Chile, Talca, Chile; 9 Department of Pathology, Asan Medical Center, University of Ulsan College of Medicine, Seoul, Korea; 10 Laboratory for Cytokine Regulation, Center for Integrative Medical Science (IMS), RIKEN Yokohama Institute, Kanagawa, Japan; 11 Institute of Hematology, Union Hospital, Tongji Medical College, Huazhong University of Science and Technology, Wuhan, People’s Republic of China; 12 Department of Paediatrics and Adolescent Medicine, LKS Faculty of Medicine, The University of Hong Kong, Hong Kong, People’s Republic of China; 13 Department of Immunology, School of Medicine, Yangtze University, Jingzhou, Hubei Province People’s Republic of China; 14 Clinical Molecular Immunology Center, School of Medicine, Yangtze University, Jingzhou, Hubei Province People’s Republic of China; 15 Center for Advanced Measurement Science, National Institute of Metrology, Beijing, People’s Republic of China; 16 Department of Respiratory, The First Affiliated Hospital of Yangtze University, Jingzhou, Hubei Province People’s Republic of China

**Keywords:** Immunological disorders, Adaptive immunity

## Abstract

The SARS-CoV-2 infection causes severe immune disruption. However, it is unclear if disrupted immune regulation still exists and pertains in recovered COVID-19 patients. In our study, we have characterized the immune phenotype of B cells from 15 recovered COVID-19 patients, and found that healthy controls and recovered patients had similar B-cell populations before and after BCR stimulation, but the frequencies of PBC in patients were significantly increased when compared to healthy controls before stimulation. However, the percentage of unswitched memory B cells was decreased in recovered patients but not changed in healthy controls upon BCR stimulation. Interestingly, we found that CD19 expression was significantly reduced in almost all the B-cell subsets in recovered patients. Moreover, the BCR signaling and early B-cell response were disrupted upon BCR stimulation. Mechanistically, we found that the reduced CD19 expression was caused by the dysregulation of cell metabolism. In conclusion, we found that SARS-CoV-2 infection causes immunodeficiency in recovered patients by downregulating CD19 expression in B cells via enhancing B-cell metabolism, which may provide a new intervention target to cure COVID-19.

## Introduction

At the end of 2019, a new coronavirus (SARS-CoV-2) was identified as the causative pathogen of the severe acute respiratory infection named COVID-19. Patients infected with SARS-CoV-2 present vastly different clinical manifestations ranging from asymptomatic forms to life-threatening pathologies including Acute Respiratory Distress Syndrome (ARDS). Evidence suggests that the so-called “cytokine storm”, uncontrolled activation of the inflammatory response, significantly contributes to the occurrence of ARDS. Similar to other diseases in which a cytokine storm is triggered, dampening of the inflammatory immune response may improve the outcome of a severe SARS-CoV-2 infection.^1,2^

Inflammation is associated with increased glycolytic activity^3^ and is the primary metabolic pathway engaged by different activated immune cells.^4,5^ Lactate as the end product of glycolysis is secreted by immune cells and elevated blood lactate and slow lactate clearance are associated with increased mortality in sepsis patients.^6^ Lactate is not only a metabolic end product, but can also be taken up by various cell types and affect both signaling and metabolism.^3^ Therefore, the hyperactive immune response leads to an altered metabolic serum composition potentially affecting further immune responses. Moreover, hepatic dysfunction and other processes can contribute to metabolic abnormalities during viral infection. Emerging evidence suggests that the metabolic composition of serum from COVID-19 patients is significantly altered in comparison to healthy individuals and that specific changes correlate with disease severity.^7^ Among other biochemicals, the levels of the TCA cycle component malate, the urea cycle component carbamoyl phosphate, and guanosine monophosphate have been suggested to change with disease progression.^8^ How immune cells and B cells in particular respond to these metabolic changes is currently unknown. However, accumulating evidence suggests that the metabolic environment plays a major role in directing B-cell fate and function.^9^ Changes in glucose concentration, access to short-chain fatty acids, oxygen availability, and other metabolic alterations profoundly affect B-cell survival, activation, and differentiation.^9^ Thus, it is conceivable that the specific metabolic environment in COVID-19 patients alters B-cell biology.

The role of B cells in COVID-19 disease progression appears to be multifaceted and is not fully understood yet. It has been recently reported that agammaglobulinemia patients who lack B cells have a more favorable disease outcome, whereas patients with common variable immune deficiency (CVID) whose B cells are dysfunctional present with a more severe form of the disease.^10^ Although the number of patients in this study was limited, the observation suggests that B cells may worsen the disease in the acute phase of the infection possibly by aggravating inflammation. On the other hand, specific B-cell subsets may play a protective role during the initial phases of infection by producing protective natural antibodies. Lastly, the formation of memory B cells and long-lived plasma cells is a crucial factor determining the level of protection in the case of a repeated virus encounter.

The goal of this study is to assess the signaling properties of B cells from COVID-19 patients. Moreover, we seek to analyze the metabolic composition of the serum obtained from COVID-19 patients and to determine how changes in the abundance of specific metabolites affect B-cell receptor signaling. We found that the cytokine milieu and the metabolic environment in COVID-19 patients shape the signaling of the B-cell population.

## Results

### SARS-CoV-2 infection alters the immune phenotype and function of B cells in recovered COVID-19 patients

**Fig. 1:**
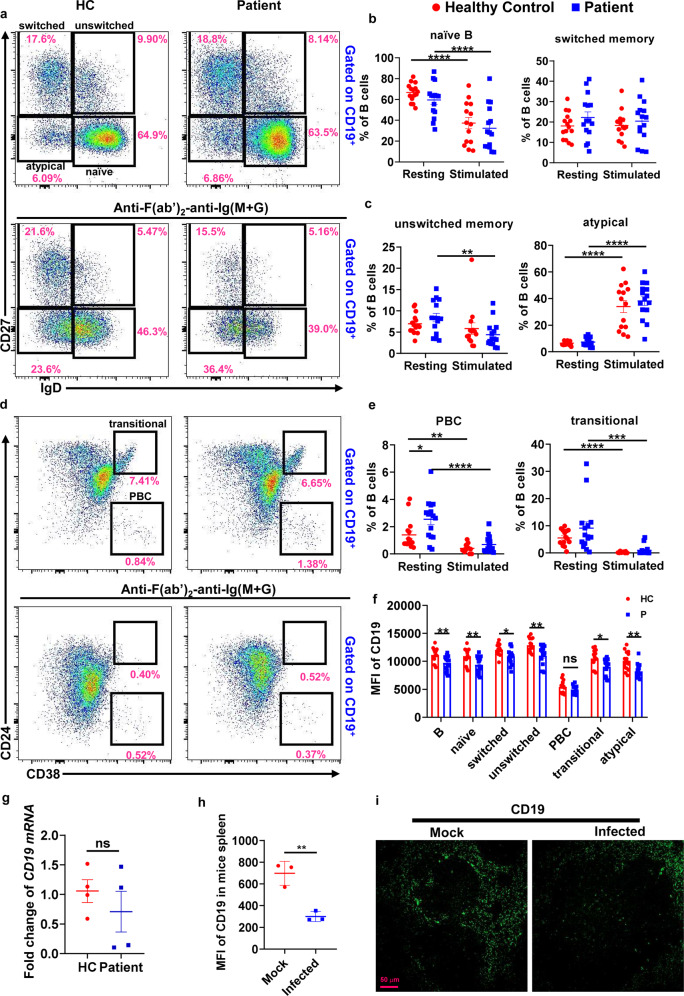
SARS-CoV-2 infection alters the immune phenotype and function of B cells and reduces CD19 expression on B cells from recovered COVID-19 patients. Flow cytometry analysis of the frequencies of naive B cells (naive, CD19^+^ IgD^+^CD27^−^), class-switched B cells (switched, CD19^+^ IgD^−^CD27^+^), unswitched memory B cells (unswitched, CD19^+^IgD^+^CD27^+^), transitional B cells (transitional, CD19^+^CD24^+^CD38^+^), plasma blasts (PBC, CD19^+^CD38^+^CD24^−^) and atypical memory B cells (atypical, CD19^+^IgD^−^CD27^−^) in resting and stimulated PBMCs from COVID-19 recovered patients (*n* = 14–15) and healthy controls (*n* = 15). Shown are representative dot plots (**a**, **d**) and the average percentages (±SEM) of B-cell subpopulations (**b**, **c**, **e**). Flow cytometry analysis of the MFI of CD19 on total B cells (B, CD19^+^), naive B cells, class-switched B cells, unswitched memory B cells, transitional B cells, plasma blasts, and atypical memory B cells in resting PBMCs from COVID-19 recovered patients (*n* = 15) and healthy controls (*n* = 15). Resting = cells before stimulation, Stimulated = cells after stimulation with 10 µg/ml biotin-F(ab′)_2_-anti-Ig(M + G) for 24 h (**f**). RT-PCR analysis of *CD19* mRNA levels of B cells from recovered patients (*n* = 4) and healthy controls (*n* = 4) (**g**). Immunofluorescence analysis of CD19 expression in nephritic sections from SARS-CoV-2 infected mice (*n* = 3) and mock mice (*n* = 3). Shown are representative images (**h**, **i**). Statistical evaluation was performed using the two-tailed Student’s *t*-test. Statistical significance is indicated using asterisks: *p < 0.05,**p < 0.01, ***p < 0.001, and ****p < 0.0001

Specific B-cell subsets play a key role in antiviral humoral immunity. To investigate whether SARS-CoV-2 infection affects the B-cell immune phenotype, we first examined the naive B cells (CD27^−^IgD^+^), atypical (CD27^−^IgD^−^), switched (CD27^+^IgD^−^) and unswitched (CD27^+^IgD^+^) memory B cells, transitional B cells (CD38^+^CD24^hi^), and plasma blast cells (PBC, CD38^+^CD24^−^) in PBMCs from recovered COVID-19 patients and healthy controls. At the resting stage, healthy control and recovered patients had similar B-cell populations, but the frequencies of PBC in patients were significantly increased when compared to healthy controls. To examine the B-cell response to BCR-dependent signaling, B cells were stimulated with F(ab′)_2_ anti-human Ig(M + G) antibodies for 24 h. The frequencies of stimulated naive B cells, PBC, and transitional B cells in both patients and healthy controls were decreased, but increased for atypical memory B cells when compared to the resting state (Fig. 1a–e). The frequencies of stimulated unswitched memory B cells in patients were decreased, but there’s no change in healthy controls when compared to the resting state (Fig. 1a, c). Interestingly, the expression of CD19, a critical regulator of BCR signaling, on total B cells and almost all B-cell subsets in recovered patients was significantly decreased compared to that of healthy controls (Fig. 1f). However, the *CD19* mRNA expression of B cells in recovered patients had no change compared to that of healthy controls (Fig. 1g). Using immunofluorescence experiments, CD19 expression was significantly reduced in spleens of SARS-CoV-2 infected mice when compared to that of non-infected (mock) mice (Fig. 1h, i). These results indicate that SARS-CoV-2 infection may alter the immune phenotype and function of B cells through inhibition of CD19 expression.

### SARS-CoV-2 infection alters the BCR signaling and B-cell metabolism in recovered COVID-19 patients

**Fig. 2:**
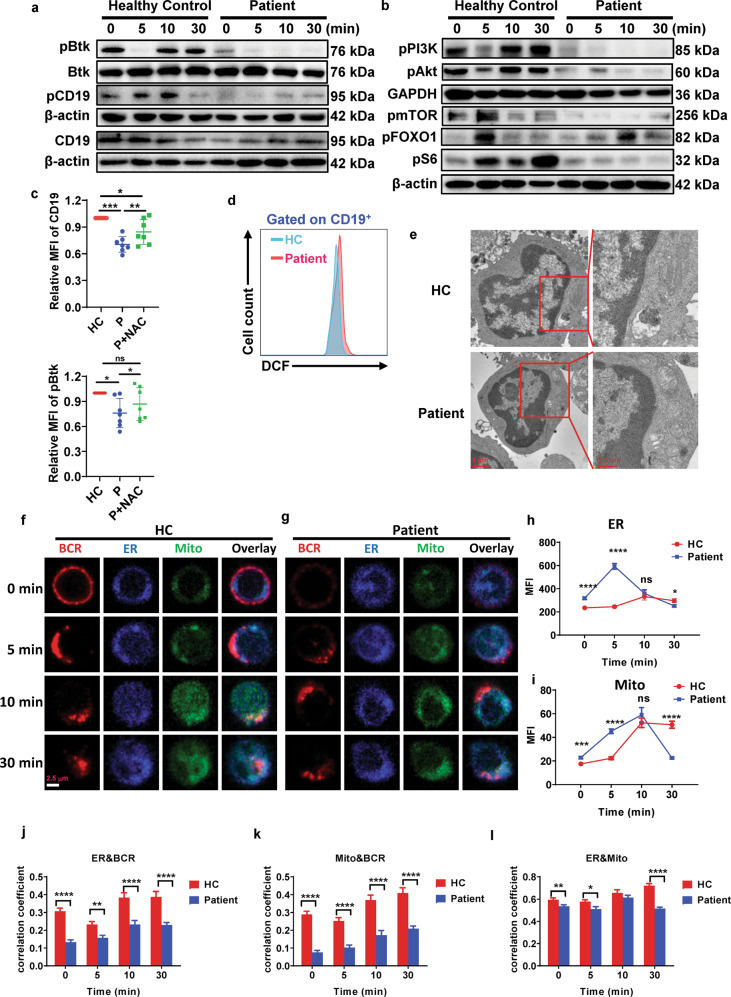
SARS-CoV-2 infection alters the BCR signaling and B-cell metabolism in recovered COVID-19 patients. B cells from COVID-19 recovered patients and healthy controls were stimulated with 10 µg/ml biotin-F(ab′)_2_-anti-Ig(M + G) plus 20 μg/ml streptavidin for indicated times, and the levels of Btk, pBtk, pCD19, CD19, pPI3K, pAkt, pmTOR, pS6, and pFoxO1 were assessed by Western blot. GAPDH or β-actin as controls. Shown are representative blots of three independent experiments (**a**, **b**). Flow cytometry analysis of the MFI of CD19 and pBtk on activated B cells for 5 min from COVID-19 recovered patients (*n* = 7) and healthy controls (*n* = 7) cultured with or without NAC (**c**). Statistical evaluation was performed using the two-tailed Student’s *t*-test. PBMCs from COVID-19 recovered patients and healthy controls were stimulated with 10 µg/ml biotin-F(ab′)_2_-anti-Ig(M + G) for 30 min, and ROS production was measured in CD19^+^ B cells (**d**). The mitochondria in B cells from COVID-19 recovered patients and healthy controls were analyzed with TEM. Shown are representative images of three independent experiments from at least 40 B cells (**e**). Scale bars, 1 μm and 500 nm. B cells from COVID-19 recovered patients and healthy controls labeled with 10 µg/ml AF594-F(ab′)_2_-anti-Ig(M + G) were activated at 37 °C for indicated times, fixed, permeabilized, and stained with probes specific for ER and mitochondria. Confocal analysis of ER and mitochondria in B cells. Shown are representative images (**f**, **g**), the MFI of ER (**h**) and mitochondria (**i**), and the correlation coefficients of BCR&ER (**j**), BCR & mitochondria (**k**), and ER& mitochondria (**l**) from three independent experiments. Scale bars, 2.5 μm. Statistical evaluation was performed using the two-tailed Student’s *t*-test. Statistical significance is indicated using asterisks: *p < 0.05,**p < 0.01, ***p < 0.001, and ****p < 0.0001

CD19 is a critical regulator of BCR signaling, B-cell development, and humoral immune response. To investigate whether SARS-CoV-2 infection affects BCR signaling, B cells from patients and healthy controls were stimulated with biotin-F(ab′)_2_ anti-human Ig(M + G) and streptavidin for indicated times, stained with antibodies specific for CD19, phosphorylated CD19, and the downstream signal protein-phosphorylated Btk, and analyzed by Western blotting. We found that the levels of total CD19, phosphorylated CD19, and phosphorylated Btk in B cells from patients were significantly decreased compared to that of healthy controls (Fig. 2a). CD19 is an important activator of the PI3K signaling pathway in anti-Ig(M + G) stimulated cells. Next, anti-Ig(M + G) stimulated B cells were examined for activation of PI3K and its downstream signals by Western blotting. Not surprisingly, we found that the phosphorylated PI3K, Akt, FoxO1, S6, and mTOR in B cells from patients were significantly decreased compared to that of healthy controls (Fig. 2b).

Because PI3K signaling is a major regulator of B-cell metabolism and redox signaling,^11^ we speculate that SARS-CoV-2 infection alters B-cell metabolism. Dysregulated production of reactive oxygen species (ROS) can lead to impaired survival of normal and germinal center B cells,^12,13^ and mitochondrial ROS suppress humoral immune response through reduction of CD19 expression in B cells in mice.^14^ We next examined the CD19 expression and phosphorylated Btk on B cells of patients after cultured with medium containing anti-oxidants (*N*-acetyl cysteine), and found the levels of CD19 and pBtk on B cells of patients in medium containing anti-oxidants was significantly increased compared to that of untreated patient B cells (Fig. 2c). To assess the redox state of B cells from COVID-19 patients, B cells from patients and healthy controls were stimulated with F(ab′)_2_ anti-human Ig(M + G), stained with the oxidant-sensing fluorescent probe dichlorodihydrofluorescein (DCFH), and measured the total ROS production. We found that B cells of patients produced more ROS than that of healthy controls (Fig. 2d). Mitochondrial complex III is required for hypoxia-induced ROS production and cellular oxygen sensing.^15^ We analyzed the mitochondria in B cells, and found that the swelling of mitochondria increased in B cells of patients compared to that of healthy controls (Fig. 2e). The maintenance of endoplasmic reticulum (ER) homeostasis shapes B-cell metabolism and metabolic stress responses by regulating mitochondrial calcium levels.^16^ To further investigate the effect of SARS-CoV-2 infection on B-cell metabolism, B cells from patients and healthy controls were stimulated with F(ab′)_2_ anti-human Ig(M + G) for varying times, stained with ER tracker and MITO tracker of mitochondria, and analyzed by confocal microscopy. We found that MFI of ER and MITO of patient B cell significantly increased at 0 and 5 min, but decreased at 30 min compared to that of healthy controls (Fig. 2f–i). Additionally, the colocalization between BCR and ER or MITO was significantly reduced throughout the B-cell activation in patients when compared to that of healthy controls (Fig. 2j–k). We also found that the colocalization between ER and MITO was significantly reduced at 0, 5, and 30 min in B cell of patients when compared with that in healthy controls (Fig. 2l). Overall, these results indicate that SARS-CoV-2 infection may alter B-cell metabolic activity and enhances ROS production, which in turn reduces CD19 expression and BCR-dependent signaling.

### SARS-CoV-2 infection alters the serum metabolite profiles and the transcriptome profile of B cells

**Fig. 3:**
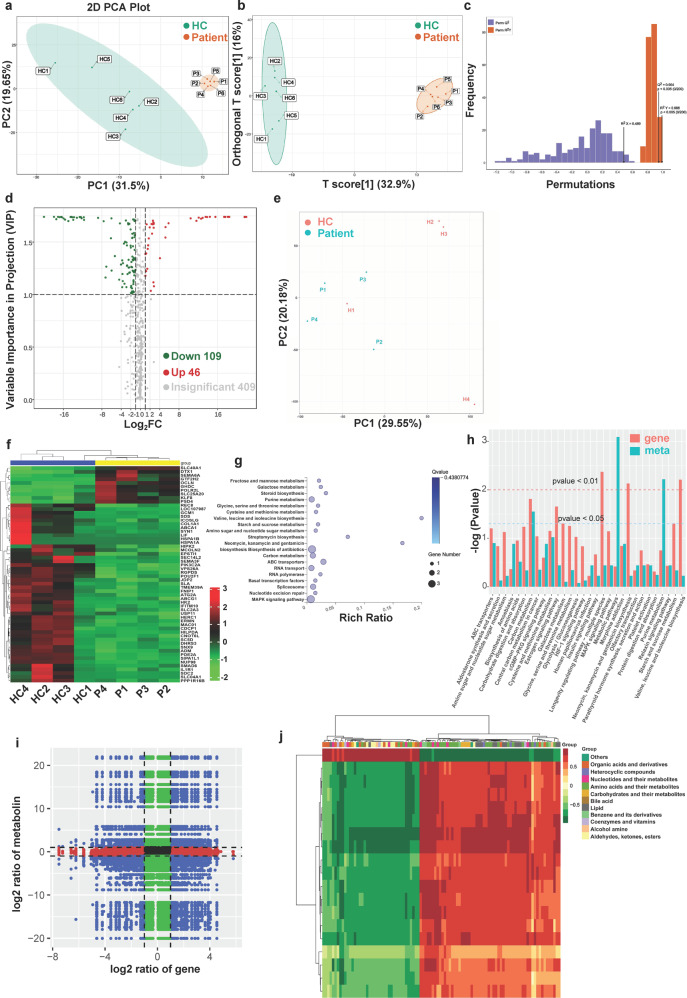
SARS-CoV-2 infection alters metabolic serum composition and the transcriptome profile of B cells to affect the BCR signaling. PCA analysis of the metabolome to intuitively show the differences between samples from COVID-19 recovered patients (*n* = 6) and healthy controls (*n* = 6) as indicated (**a**). OPLS-DA showed the possible discrimination of metabolites in serum from COVID-19 recovered patients (*n* = 6) and healthy controls (*n* = 6) as indicated. *R*^2^ and *Q*^2^ represent goodness of fit and prediction respectively, and *p*-value shows the significance level of the model (*x* axis = predictive components, *y* axis = orthogonal component) (**b**, **c**). By combining FC with VIP from the OPLS-DA model, the volcano plot showed different abundant metabolites in serum from COVID-19 recovered patients. Two clusters consisting of 46 up- and 109 downregulated metabolites. Significantly differentially abundant metabolites were color-coded: red indicated upregulated proteins, green showed downregulated proteins (**d**). PCA analysis of the transcriptome to intuitively show the differences between samples from COVID-19 recovered patients (*n* = 4) and healthy controls (*n* = 4) as indicated (**e**). Clustering heat map of the differentially expressed genes in B cells from 4 healthy controls and 4 COVID-19 recovered patients. The horizontal axis represents the sample and the longitudinal axis represents the differentially expressed genes. Red = positive correlation, green = negative correlation (**f**). The KEGG pathway analysis of genes in recovered COVID-19 patients and controls. The color and the size of bubbles represent respectively the *p*-value and the number of counts (**g**). KEGG enrichment analysis of the differentially abundant pathways by combing the differentially abundant metabolites with the differentially expressed genes. The name of the pathway as shown on the bottom; The higher the height of the column, indicated a smaller *p*-value (**h**). The nine-quadrant plot shows the different multiples of genes and metabolites with a Pearson’s correlation coefficient >0.8 in each differential group with black dotted lines (**i**). For the differential metabolites and genes with a correlation coefficient of more than 0.8 in the above analysis, the correlation coefficient clustering heat map shows the selected correlation results (**j**)

To investigate the differences in the metabolic compositions of serum between recovered COVID-19 patients and healthy controls, we performed a metabolic analysis. Principal component analysis (PCA) was performed on the metabolome and the orthogonal partial least-squares discriminant analysis (OPLS-DA) was used to discriminate metabolic profiles between the groups of recovered COVID-19 patients and healthy controls (Fig. 3a–c). By combining FC with VIP from the OPLS-DA model, 155 metabolites, including 109 downregulated and 46 upregulated were finally altered in the serum of recovered COVID-19 patients (Fig. 3d). Among the altered metabolites, the levels of the amino acids l-arginine, l-glutamic acid, l-isoleucine, l-cystine, and l-cysteine were significantly reduced in the serum from recovered COVID-19 patients. To validate the results from omics data, the levels of glutamic and cysteine in serum were measured, and we found the levels of glutamic and cysteine in serum from recovered COVID-19 patients were significantly decreased compared to that of healthy controls (Supplemental Fig. 1). Collectively, these results provide a rationale to explore how serum metabolic components alter B-cell signaling and function.

We detected the transcriptome differences of B cells between recovered COVID-19 patients and healthy controls. PCA was performed on the transcriptome, and shown the differences between the healthy controls and recovered COVID-19 patients (Fig. 3e). The expression of 48 genes was significantly downregulated and 10 genes were significantly upregulated when comparing recovered COVID-19 patients versus healthy controls (Fig. 3f). Then, we performed the Kyoto Encyclopedia of Genes and Genomes (KEGG) functional enrichment analysis, and found that differentiating genes/metabolic genes enriched in a total of 20 pathways, most of which were metabolic signaling pathways, especially amino acid synthesis and metabolism (Fig. 3g). Finally, to investigate the differences of the genes related to metabolism and metabolism products, a combined analysis of the transcriptome and metabolome was performed. We found the genes related to metabolism and metabolism products were dysregulated. KEGG was used to identify metabolic pathways of genes and metabolites, 30 metabolic pathways were found significantly different between healthy controls and recovered COVID-19 patients (Fig. 3h). In the transcriptome, degrees of enrichment was higher in the Longevity regulating pathway-multiple species, valine, leucine, and isoleucine biosynthesis, Neomycin, kanamycin, and gentamicin biosynthesis, carbon metabolism, and estrogen signaling pathway (Fig. 3h). In the metabolome, degrees of enrichment was higher in purine metabolism and carbon metabolism (Fig. 3h). Correlation analysis was performed for genes and metabolites detected in each differential grouping. The nine-quadrant diagram and Heat map clustering were used to show the different multiples of the metabolites in each differential group with Pearson’s correlation coefficient >0.8. We found that the hypermetabolic state in the recovered patients group (Fig. 3i, j). Moreover, to validate the results from omics data, the mRNA levels of several genes shown in Fig. 2f in B cells were measured by RT-PCR, and we found that mRNA levels of *HK2*, *SLC04A1*, *SDS*, and *COL1A1* were significantly decreased in B cells of COVID-19 patients compared to that of healthy controls (Supplemental Fig. 2). These results suggest that SARS-COV-2 may alter B-cell metabolism through these pathways.

In summary, SARS-CoV-2 infection may affect the BCR signaling by altering the metabolomic and transcriptome profiles of B cells.

### SARS-CoV-2 infection alters the BCR signaling through affecting early activation of B cell

**Fig. 4:**
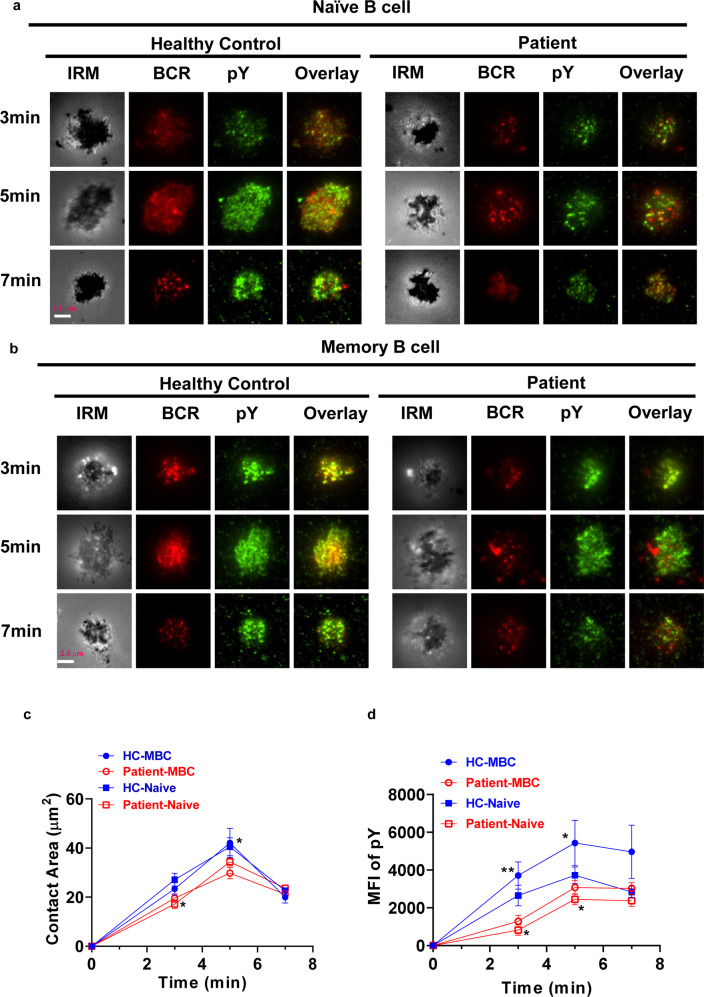
SARS-CoV-2 infection alters the BCR signaling through affecting the B-cell early activation. Naive (CD27^−^) and memory (CD27^+^) B cells from COVID-19 recovered patients and healthy controls were activated with 10 µg/ml AF546–mB-Fab′–anti-Ig tethered to lipid bilayers at 37 °C for 3, 5 and 7 min. Then cells were fixed, permeabilized, and stained with Alexa Fluor 647 anti-CD27 and anti-pY (**a**, **b**), followed by Alexa Fluor 488 goat anti-mouse IgG. Cells were analyzed by Tirf microscope. Shown are representative images, the mean values of the B-cell contact area (**c**), and the MFI of pY (**d**) in the contact zone from three independent experiments. Scale bars, 2.5 μm. Statistical evaluation was performed using the two-tailed Student’s *t*-test. Statistical significance is indicated using asterisks: *p < 0.05,**p < 0.01, ***p < 0.001, and ****p < 0.0001

**Fig. 5:**
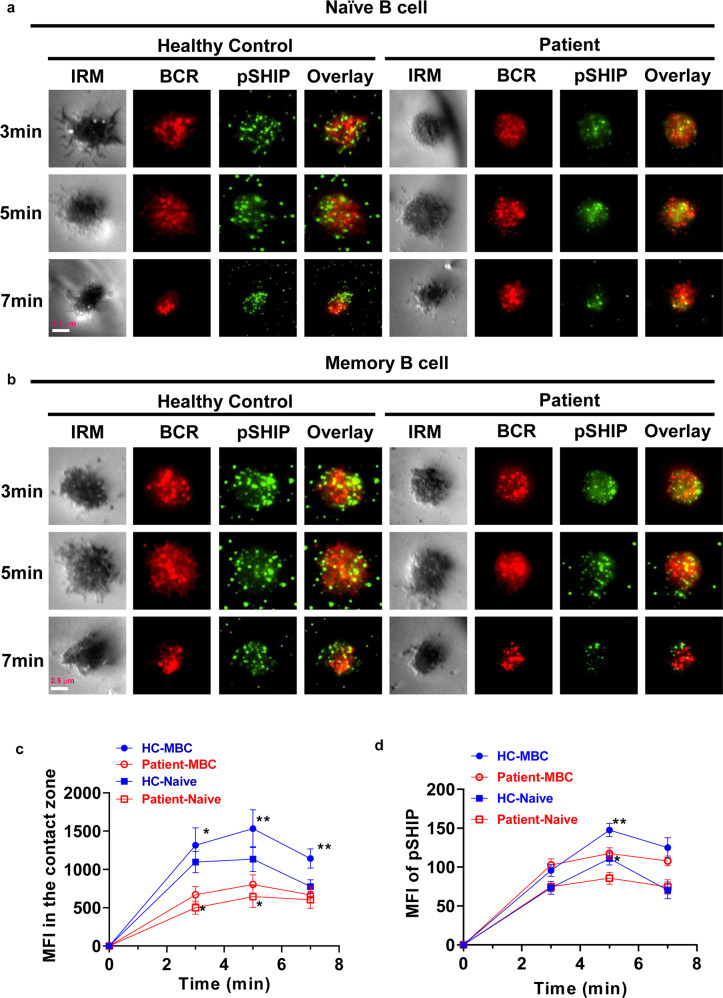
SARS-CoV-2 infection inhibits the early activation of SHIP in B cells. Naive (CD27^−^) and memory (CD27^+^) B cells from COVID-19 recovered patients and healthy controls were activated with 10 µg/ml AF546–mB-Fab′–anti-Ig tethered to lipid bilayers at 37 °C for 3, 5 and 7 min. Then cells were fixed, permeabilized, and stained with Alexa Fluor 647 anti-CD27 and anti-pSHIP (**a**, **b**), followed by Alexa Fluor 488 goat anti-rabbit IgG. Cells were analyzed by Tirf microscope. Shown are representative images, and the MFI of BCR (**c**) and pSHIP (**d**) in the contact zone from three independent experiments. Scale bars, 2.5 μm. Statistical evaluation was performed using the two-tailed Student’s *t*-test. Statistical significance is indicated using asterisks: *p < 0.05, **p < 0.01, ***p < 0.001, and ****p < 0.0001

**Fig. 6:**
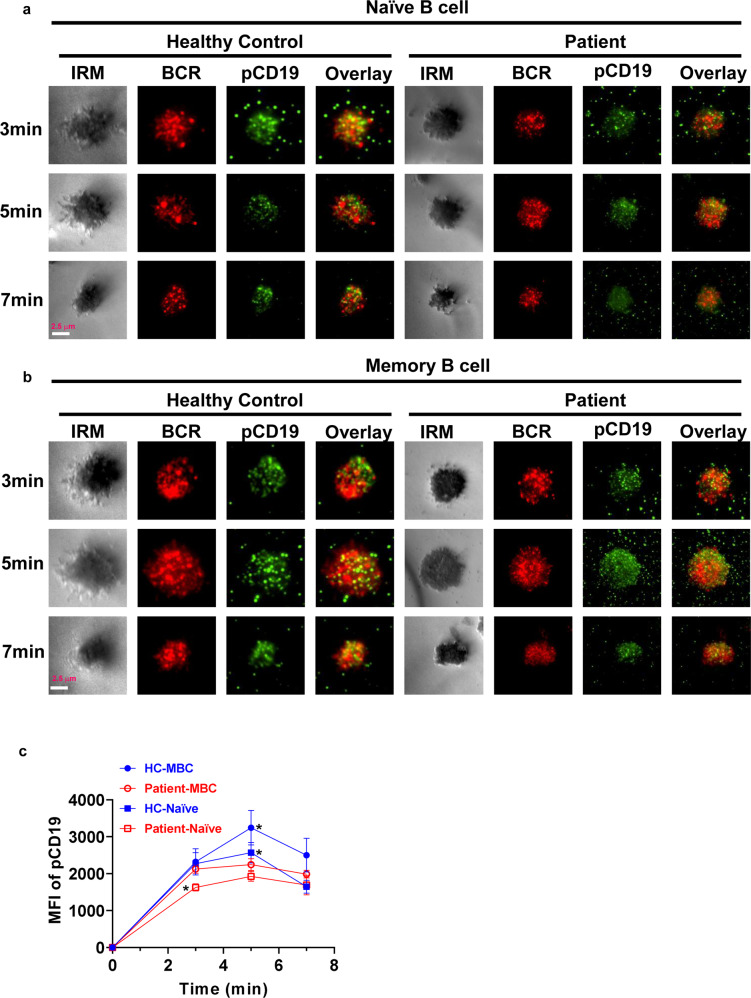
SARS-CoV-2 infection inhibits the early activation of CD19 in B cells. Naive (CD27^−^) and memory (CD27^+^) B cells from COVID-19 recovered patients and healthy controls were activated with 10 µg/ml AF546–mB-Fab′–anti-Ig tethered to lipid bilayers at 37 °C for 3, 5 and 7 min. Then cells were fixed, permeabilized, and stained with Alexa Fluor 647 anti-CD27 and anti-pCD19 (**a**, **b**), followed by Alexa Fluor 488 goat anti-rabbit IgG. Cells were analyzed by Tirf microscope. Shown are representative images and the MFI of pCD19 (**c**) in the contact zone from three independent experiments. Scale bars, 2.5 μm. Statistical evaluation was performed using the two-tailed Student’s *t*-test. Statistical significance is indicated using asterisks: *p < 0.05, **p < 0.01, ***p < 0.001, and ****p < 0.0001

To investigate the effect of SARS-CoV-2 infection on early activation of B cells and BCR signaling, TIRFm was applied to observe the B-cell spreading, BCR clustering, signalosome accumulation, and BCR signaling. B cells from patients and healthy controls were stimulated with membrane-associated antigen for varying lengths of time and then stained for pCD19 and phospho-Tyrosines (pY) with specific antibodies. AF647-CD27 was used to distinguish naive (CD27^−^) and memory (CD27^+^) B cells. B-cell spreading was shown with the contact area, and BCR clustering was measured using MFI. For B-cell spreading, contact area was reduced in naive B cells of patients at 3 min, and significantly decreased in memory B cells of patients at 5 min after activation compared with that in naive and memory B cells of healthy controls (Fig. 4a–c). For total BCR signals, MFI of pY was significantly decreased at 3 and 5 min in naive B cells of patients when compared to that of naive and memory B cells of healthy controls (Fig. 4a, b, d). For BCR clustering, MFI of BCRs was significantly reduced at 3 and 5 min both in naive and memory B cells of patients when compared to that of naive and memory B cells of healthy controls (Fig. 5a–c). Moreover, the MFI of pSHIP, one negative BCR signal, was significantly reduced at 5 min in naive and memory B cells after activation compared with that of naive and memory B cells of healthy controls (Fig. 5a, b, d). For activation of CD19, MFI of pCD19 was significantly reduced at 3 and 5 min in naive and memory B cells of patients when compared to that in naive and memory B cells of healthy controls (Fig. 6a–c). These results indicate that SARS-CoV-2 infection alters the BCR signaling through inhibiting the early activation of naive and memory B cells.

## Discussion

The SARS-CoV-2 infection has caused immune disruption according to previous reports. Studies have reported that COVID-19 patients had decreased lymphocyte counts and increased counts of myeloid cells in peripheral blood.^17,18^ Several recent reports show lymphopenia with drastically reduced numbers of both CD4^+^ and CD8^+^ T cells in moderate and severe COVID-19 cases.^19,20^ Flow cytometry analysis of PBMCs from symptomatic COVID-19 patients has shown a significant influx of granulocyte-macrophage colony-stimulating factor (GM-CSF)-producing, activated CD4^+^ T cells and CD14^+^ HLA-DR^lo^ inflammatory monocytes (IMs).^21,22^ Multiple studies have shown reduced numbers of NK cells in the PBMC of COVID-19 patients, which is associated with the severity of the disease.^20,23^ However, it is unclear if the disrupted immune regulation still exists and pertains in recovered patients. In our study, we have characterized the immune phenotype of B cells from 15 recovered COVID-19 patients. Healthy control and recovered patients had similar B-cell populations at the resting and stimulated state. However, the frequencies of PBC in patients were significantly increased when compared to healthy controls before stimulation, and the percentage of unswitched memory B cells was decreased in recovered patients but not changed in healthy controls upon BCR stimulation. More interestingly, we found that CD19 expression was significantly reduced in almost all the B-cell subsets in recovered patients. CD19 is one of the members of the B-cell receptor co-complex, consisting of CD19, CD81, CD21, and CD225, which effectively amplifies antigenic signaling via the B-cell receptor (BCR). Thus, although the CD19 expression in recovered COVID-19 patients was mildly reduced within a range of about 10% probably, BCR signaling was largely decreased in B cells from recovered patients upon BCR activation. CD19 is a critical regulator of BCR signaling, B-cell development, and humoral immune response. We previously reported that CD19 downregulation leads to immunodeficiency by regulating the early activation of memory B cells in Wiskott–Aldrich syndrome patients.^24^ Not surprisingly we found that the BCR signaling and early B-cell response including B-cell spreading, BCR clustering, and signalosome recruitment was disrupted upon BCR stimulation in recovered patients. Mechanistically, the reduced CD19 expression is caused by enhanced ROS production, instead of gene transcription, in recovered COVID-19 patients. Together, we have established the immune deficiency in recovered patients is due to the reduced CD19 expression, which dampens the BCR signaling.

Accumulating evidence suggests that the metabolic environment plays a major role in directing B-cell fate and function, and changes in oxygen availability and other metabolic alterations profoundly affect B-cell survival, activation, and differentiation.^4,9^ In our study, among the altered metabolites in the analysis of serum metabolite profiles, the levels of the amino acids l-arginine, l-glutamic acid, l-isoleucine, l-cystine, and l-cysteine were significantly reduced in the serum from recovered COVID-19 patients. These results indicate that the disrupted metabolism may affect B-cell activation and function in recovered patients.

Moreover, it’s reported that increased mitochondrial ROS production has been shown to result in decreased CD19 expression,^14^ which reduces BCR signaling. In our study, ROS was found to increase and CD19 expression was decreased, and interestingly, CD19 expression and BCR signaling was rescued partially in recovered patients by using ROS scavenger-NAC. These results are in line with what has been reported and indicate that increased ROS may affect BCR signaling in recovered patients through inhibiting CD19 expression.

By using transcriptome and metabolism, we found the genes related to metabolism and metabolism products were dysregulated. Additionally, we found that the ROS of B cells in recovered patients was increased, which indicates an increased cell metabolism of B cells in recovered patients. Previous studies reported that metabolite and lipid alterations are shown apparent correlation with the course of disease in these COVID-19 patients, suggesting that the development of COVID-19 affected whole-body metabolism of the patients.^8,25^ Interestingly, CD19 expression was rescued partially in recovered patients by using ROS scavenger-NAC. We have demonstrated that the increase of the metabolic state causes decreased expression of the critical immune regulator-CD19 and the immunodeficiency state in recovered patients. Moreover, we found that the CD19 expression was also reduced in the spleen of infected mice with SARS-CoV-2. We can refer that CD19 expression may be reduced in infected patients as well. It could be interesting to examine the CD19 expression in mild and severely infected patients and the metabolism state of B cells. Furthermore, the correlation between the CD19 expression and the different metabolism states of B cells is worthy to pursue. More excitingly, the correction of the increased ROS in B cells rescued the CD19 expression and BCR signaling, which may correct the immune state in recovered patients. It is unknown whether the recovered patients having reduced CD19 expression are more susceptible to be re-infected by SARS-CoV-2. If it is true, the metabolism regulator such as NAC may have a potential clinical application on the prevention of second infection of recovered patients.

Although we found the decreased CD19 expression, the level of *CD19* mRNA had no change in B cells from recovered patients, which indicates that SARS-CoV-2 infection may reduce the CD19 levels in the period of post-transcriptional translation. We also checked several molecules in the BCR signaling pathway from RNA-Seq, but did not find the differences of *CD19* mRNA and molecules in the BCR signaling pathway between HC and patients. That’s the reason why the BCR signaling pathway didn’t appear in the KEGG enriched signaling pathways. The current omics data were analyzed for unstimulated B cells, however, results of the B-cell experiment in vitro were shown when the B cells were activated with F(ab′)_2_-anti-human Ig (M + G). Therefore, the omics data seem to not effectively match the results of the B-cell experiment in vitro. It would be interesting to analyze the omics data from B cells activated with F(ab′)_2_-anti-human Ig (M + G).

Bacterial and fungal infections are common complications in patients with viral pneumonia and lead to increased mortality. Analyzing the signaling properties of B cells from COVID-19 patients would help to assess whether a COVID-19 infection renders patients more susceptible to other diseases. Moreover, a better understanding of how metabolic abnormalities observed in patient serum affect B-cell signaling could inspire the development of new treatment strategies targeting specific metabolic pathways. Different compounds altering cell metabolism and the redox state are currently tested in pre-clinical and clinical studies^26–28^ to treat autoimmune disorders and could also be exploited to dampen an overactive immune response during a viral infection.

## Materials and methods

### Patients and PBMCs collection

COVID-19 patients were recruited from the Central Hospital of Wuhan, Wuhan, China. These subjects conformed to the diagnostic criteria according to the fifth edition of guidelines of diagnosis and treatment of COVID-19, Chinese National Health Commission, and were discharged after two consecutively negative SARS-CoV-2 nucleic acid tests through RT-PCR. Healthy donors with negative SARS-CoV-2 nucleic acid and specific IgM and IgG antibodies were also recruited from the Central Hospital of Wuhan, China. Peripheral blood samples were collected from COVID-19 recovered patients (10–12 weeks after original diagnosis) and healthy controls. Peripheral blood mononuclear cells (PBMCs) were isolated using Ficoll (GE Ficoll-Paque PLUS, 17144002) according to the standard procedure. The cell viability in each sample exceeded 85%. Plasma samples were stored at −80 °C. Written informed consents were provided from all subjects with the approval of the Ethics Committee of the Huazhong University of Science and Technology.

### SARS-CoV-2, cell culture, and virus titrations

SARS-CoV-2 (IVCAS 6.7512) was obtained and isolated from the bronchoalveolar lavage fluid collected of a COVID-19 patient. Vero E6 cells (ATCC®CRL-1586™) were used to propagate SARS-CoV-2 in DMEM containing 10% FBS, 1 mM l-glutamine, as well as 1% streptomycin–penicillin, and cultured at 37 °C in 5% CO_2_. The cell supernatant was centrifuged and harvested on the 3rd day after infection. Vero E6 cells were incubated with 10-fold serial dilutions of cell supernatant. One hour later, the medium was dropped and replaced with new DMEM supplemented with 2% FBS, 1 mM l-glutamine, and 1% streptomycin–penicillin. Three days after inoculation, cytopathic effect (CPE) was scored and the TCID_50_ was calculated as described previously.^29^

### Mice and infection

The human ACE2 protein (hACE2) transgenic mice (HFH4-hACE2 mice) on the mixed genetic backgrounds (C3H, C57BL/6) were donated kindly by Z.Shi’s laboratory, as been described previously.^30,31^

The mice were anesthetized with tribromoethanol (250 mg/kg) and then intranasally inoculated with 3*10^4^ TCID_50_ SARS-CoV-2 in 50 μl DMEM per mouse as described before.^31^ Mice in the mock group were administered with an equal volume of DMEM. Mice were euthanized at 7 days post-infection, and spleens were harvested. Viral infections were performed in a biosafety level 3 (BSL-3) facility in accordance with guidelines of the Institutional Animal Care and Ethics Committee of Animal Experimentation and the Institutional Review Board of the Wuhan Institute of Virology, CAS (ethics number WIVA05202003).

### Flow cytometry

PBMCs from COVID-19 recovered patients and healthy donors were stained with specific antibodies as follows: FITC-anti-CD19 (302206, Biolegend), Pacific Blue-anti-CD38 (356628, Biolegend), PE-anti-CD24 (311106, Biolegend), Alexa Fluor 647 anti-CD27 (302812, Biolegend), and Brilliant Violet 510-anti-IgD (348220, Biolegend). For B-cell transformation analysis, PBMCs from COVID-19 recovered patients and healthy donors were incubated in complete medium with 10 μg/ml biotin-F(ab′)_2_ anti-human Ig(M + G) (109-066-127, Jackson) in 5% CO_2_ for 24 h. Cells were collected and stained with as previously described on ice for 30 min. After being washed twice by PBS, samples were collected and analyzed by a multicolor flow cytometer (Attune NxT, AFC2, ThermoFisher). Data were analyzed using the FlowJo software (TreeStar, USA).

### ROS inhibition and phosflow

PBMCs from COVID-19 recovered patients and healthy donors were incubated in a complete medium with or without 10 mM NAC (A7250-50G, Sigma-Aldrich) in 5% CO_2_ for 24 h. Cells were collected and stained with PE-anti-CD19 (302208, Biolegend), then cells were incubated with 10 μg/ml biotin-F(ab′)_2_ anti-human Ig(M + G) on ice for 30 min, plus 20 μg/ml streptavidin on ice for 10 min, and then activated at 37 °C for 5 min. After being fixed with 4% paraformaldehyde, cells were permeabilized with 0.05% saponin (S4521-10G, Sigma-Aldrich), and stained with anti-pBtk (ab52192, Abcam, USA), following by Alexa Fluor 647 goat anti-rabbit IgG (A-21245, ThermoFisher, USA). Samples were analyzed by flow cytometer.

### Western blot

PBMCs from COVID-19 recovered patients and healthy donors were incubated with 10 μg/ml biotin-F(ab′)_2_ anti-human Ig(M + G) on ice for 30 min, plus 20 μg/ml streptavidin on ice for 10 min, and then activated at 37 °C for indicated times. Cell lysates were used for electrophoresis in SDS–polyacrylamide gel, electrotransferred onto a nitrocellulose membrane, and then probed with the following specific antibodies: anti-pCD19 (Cat# 3571S, Cell Signaling Technology, USA), anti-CD19 (Cat# 90176, Cell Signaling Technology, USA), anti-pBtk (Cat# ab52192, Abcam, USA), anti-Btk (Cat# 8547S, Cell Signaling Technology, USA), anti-pPI3K (Cat# 4228S, Cell Signaling Technology, USA), anti-pAkt (Cat# 4060L, Cell Signaling Technology, USA), anti-pFoxO1 (Cat# 9461S, Cell Signaling Technology, USA), anti-pmTOR (Cat# 5536S, Cell Signaling Technology, USA), and anti-pS6 (Cat# 4856S, Cell Signaling Technology, USA). After incubated relative secondary antibodies, immunoreactive bands were presented and captured with the ChemiDoc XRS + imaging systems (Bio-Rad). β-actin or GAPDH was used as the loading control.

### Confocal and total internal reflection fluorescence microscope (TIRFm)

For confocal analysis, purified B cells from COVID-19 recovered patients and healthy donors were stained with 10 μg/ml with Alexa Fluor 594-F(ab′)_2_ anti-human Ig(M + G) (109-586-127, Jackson) (sAg) on ice for 30 min and activated for 0, 5, 10, and 30 min at 37 °C. After stimulation, B cells were fixed with 4% paraformaldehyde, permeabilized with 0.05% saponin (S4521-10G, Sigma-Aldrich), and stained with AF405-ER-Tracker dyes (E12353, Invitrogen, USA) and AF647-probes for mitochondria (M7512, Invitrogen, USA). Images were captured using a confocal microscope (Nikon) with 405-, 488-, and 546-nm lasers. MFI and colocalizations were determined using the NIS-elements AR 5.01.

For TIRFm analysis, B cells in PBMCs from COVID-19 patients and healthy controls were activated with 10 µg/ml AF546–mB-Fab′–anti-Igtethered to lipid bilayer at 37 °C for varying lengths of time as described before.^32^ Then cells were fixed with 4% paraformaldehyde, permeabilized with 0.5% saponin, and stained with Alexa Fluor 647 anti-CD27 (302812, Biolegend, USA), anti-pY (05-321, Millipore, Merck, USA), anti-pCD19 (3571S, Cell Signaling Technology, USA), and pSHIP (3941S, Cell Signaling Technology, USA), followed by Alexa Fluor 488 goat anti-mouse IgG (715-165-151, Jackson, USA) and Alexa Fluor 488 goat anti-rabbit IgG (A-11008, ThermoFisher, USA). Images were captured using TIRFm (Nikon) and interference reflection microscopy (IRM). Data were analyzed using the NIS-elements AR 5.01.

### Immunofluorescence

Spleens of HFH4-hACE2 mice infected or not were fixed with 4% paraformaldehyde, embedded in paraffin, and cut into 3.5-mm sections. For immunofluorescence, splenic sections were de-paraffinized and rehydrated, followed by heat-induced antigen retrieval with EDTA pH = 8.0 for 15 min. Then samples were washed with PBS containing 0.02% Triton X-100, blocked with 5% BSA at room temperature for 1 h, and stained with FITC-CD19 (1:20; Cat# 557398, BD Bioscience) for 30 min at room temperature. Finally, an antifade reagent was added to prevent fluorescence quenching. The images were captured using a confocal microscope (Nikon) and data were analyzed with the NIS-elements AR software.

### Combined transcriptional and metabolic analysis in B cells and serum

For transcriptional analysis, B cells in PBMCs from healthy controls (*n* = 4) and recovered patients (*n* = 4) were purified by human CD19 MicroBeads (130-050-301, Miltenyi) according to standard protocols for RNA sequence analysis. For metabolic analysis, 50 μl of serum samples (healthy control group, *n* = 6; patient group, *n* = 6) were thawed on ice, and metabolites were extracted by a modified method.^33^ In short, 3 volumes of ice-cold methanol were mixed with 1 volume of serum. After whirling the mixture for 3 min and centrifugation with 12,000 rpm at 4 °C for 10 min, the supernatant was collected and centrifuged at 12,000 rpm at 4 °C for 5 min. Finally, collect the supernatant again for LC-ESI-MS/MS analysis.

The sample extracts were analyzed using an LC-ESI-MS/MS system (UPLC, Shim-pack UFLC SHIMADZU CBM A system, https://www.shimadzu.com/; MS, QTRAP® System, https://sciex.com/) according to the standard protocols.

The triple quadrupole-linear ion trap mass spectrometer (QTRAP) was used to perform LIT and triple quadrupole (QQQ) scans, which was operated and controlled by Analyst 1.6.3 software (Sciex) according to the standard parameters. After instrument tuning and mass calibration, the specific MRM transitions were examed according to the metabolites eluted within this period.

All raw LC-MS/MS data have been deposited to the iProX under the accession number: PXD018307. For unsupervised PCA (principal component analysis), unit variance scaled data was analyzed by statistics function prcomp within R (www.r-project.org). For HCA (hierarchical cluster analysis), results of metabolites were shown as heatmaps, and PCC (Pearson correlation coefficients) between samples were calculated by the cor function in R (www.r-project.org) and presented as heatmaps; For HCA, the color spectrum was applied to display normalized signal intensities of metabolites (unit variance scaling). The orthogonal projection to latent structure discriminant analysis (OPLS-DA) model was used to identify the differences in metabolic profiles between groups maximumly. For analysis of differential metabolites selected, significantly regulated metabolites between groups were determined by VIP ≥ 1 and absolute Log2FC (fold change) ≥ 1. VIP values were extracted from OPLS-DA result, which also contains score plots and permutation plots, was generated using the R package MetaboAnalystR. The data was log transform (log2) and mean centering before OPLS-DA. A permutation test (200 permutations) was applied to avoid overfitting. Last, for pathway enrichment analysis, the KEGG (Kyoto Encyclopedia of Genes and Genomes) database (http://www.genome.Jp/kegg/) to was used to find highly enriched metabolic signal transduction pathways in differential metabolites between two groups. Differences between the two groups were determined by the hypergeometric test’s, and the *p*-value < 0.05 was considered significantly changing pathways.

### ROS detection

PBMCs from COVID-19 recovered patients and healthy donors were stained with FITC-anti-CD19 (302206, Biolegend, USA) and DCFH-DA (S0033, Beyotime Biotechnology, China) on ice for 30 min. After being washed by PBS containing no FBS, PBMCs were incubated with 10 μg/ml biotin-F(ab′)_2_ anti-human Ig(M + G) on ice for 30 min, stimulated at 37 °C for 30 min, and then analyzed by flow cytometer (Attune NxT, AFC2, ThermoFisher). MFI of DCF in CD19^+^ B cells was measured using the FlowJo software (TreeStar, USA).

### Determination of cysteine and glutamic

Cysteine and glutamic contents in serum were determined by cysteine assay kit (BC0185, Beijing Solarbio Science & Technology Co., Ltd, China) and glutamic assay kit (BC1585, Beijing Solarbio Science & Technology Co., Ltd, China), respectively.

### Transmission electron microscopy (TEM)

B cells purified from PBMCs of COVID-19 recovered patients and healthy donors were fixed in 2.5% glutaraldehyde in 0.1 M phosphate buffer for 2 h at 4 °C. Samples were centrifuged and post-fixed in 2% OsO4 in cacodylate buffer, pH 7.3 for 1 h at 4 °C. Samples were dehydrated stepwise through ethanol (30%, 50%, 70%, 90%, 100%) and embedded in epon. Sections were cut on an ultramicrotome (UC7 ultramicrotome; Leica) and placed on Formvar-coated slot grids, and sections were stained with lead citrate. Sections were viewed on a transmission electron microscope (H-7000FA, HITACHI, Japan) and images were captured.

### Quantitative RT-PCR

B cells purified from PBMCs by human CD19 MicroBeads (130-050-301, Miltenyi) were used to compare the gene expression in healthy controls and recovered patients. Total cellular RNA was extracted by Trizol (15596026, ThermoFisher Scientific), and reverse transcribed into complementary DNA using PrimeScript RT Reagent Kit (RR047A, Takara). And the expression level was analyzed by CFX96 Touch equipment (Bio-Rad). The primer sequences were as follows:

*CD19*:

5′ primer: tactatggcactggctgctg

3′ primer: cacgttcccgtactggttct

hexokinase 2 (*HK2*):

5′ primer: tctatgccatccctgaggac

3′ primer: tctctgccttccactccact

solute carrier organic anion transporter family member 4A1 (*SLCO4A1*):

5′ primer: gaagctgccaccttgtttgg

3′ primer: gctctggagctggctatctg

serine dehydratase (*SDS*):

5′ primer: aggccctagcgaagaacaac

3′ primer: tcggagattcccctccagtt

collagen type I alpha 1 chain (*COL1A1*):

5′ primer: gtgctaaaggtgccaatggt

3′ primer: ctcctcgctttccttcctct

*GAPDH*:

5′ primer: acccagaagactgtggatgg

3′ primer: ttctagacggcaggtcaggt

### Statistical analysis

Unpaired two-tailed Student’s *t*-test or paired two-tailed Student’s *t*-test was used for two groups’ comparisons. Statistical differences were analyzed using the GraphPad Prism 8 Software (San Diego, CA). Data were presented as the mean and standard error of mean (SEM). The difference was considered significant when the value of *p* is below 0.05. *p < 0.05, **p < 0.01, ***p < 0.001, ****p < 0.0001.

## Supplementary information

Supplementary Materials

RAW DATA

## Data Availability

The data sets and any other raw data that support the findings of this study are available from the corresponding author upon reasonable request.
