# Supplementary material for: SARS-CoV-2 infection causes immunodeficiency in recovered patients by downregulating CD19 expression in B cells via enhancing B-cell metabolism
Source: Signal Transduct Target Ther. 2021 Sep 22;6:345. doi: 10.1038/s41392-021-00749-3 (PMC8456405; doi:10.1038/s41392-021-00749-3)
Supplement: Supplementary file 1 — Supplementary Materials [file 41392_2021_749_MOESM1_ESM.docx]

Supplementary Materials for

**SARS-CoV-2 infection causes immunodeficiency in recovered patients by downregulating CD19 expression in B cells via enhancing B cell metabolism**

Yukai Jing1, 2, 3*, Li Luo4*, Ying Chen5, Lisa S. Westerberg6, Peng Zhou5, Zhiping Xu7, Andrés A. Herrada8, Chan-Sik Park9, Masato Kubo10, Heng Mei11, Yu Hu11, Pamela Pui-Wah Lee12, Bing Zheng13,14, Zhiwei Sui15, Wei Xiao16, Quan Gong13,14, Zhongxin Lu1†, Chaohong Liu4,17†

**This PDF file includes:**

Figures S1 to S2


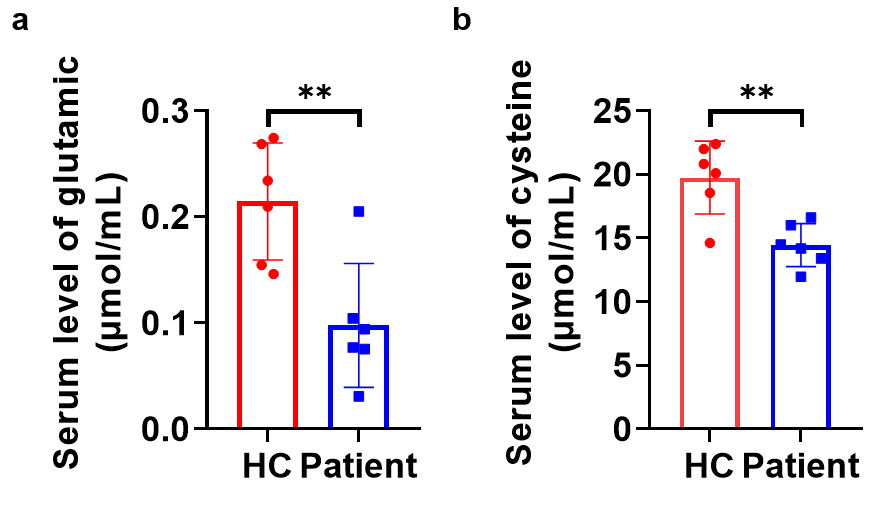


Supplemental Fig. S1. SARS-CoV-2 infection alters the serum profile of amino composition in recovered COVID-19 patients. The serum levels of glutamic and cysteine were detected by glutamic and cysteine assay kits respectively (n=6). The results were shown (a and b). Statistical evaluation was performed using the two-tailed Student’s *t*-test. Statistical significance is indicated using asterisks: ***P* < 0.01.


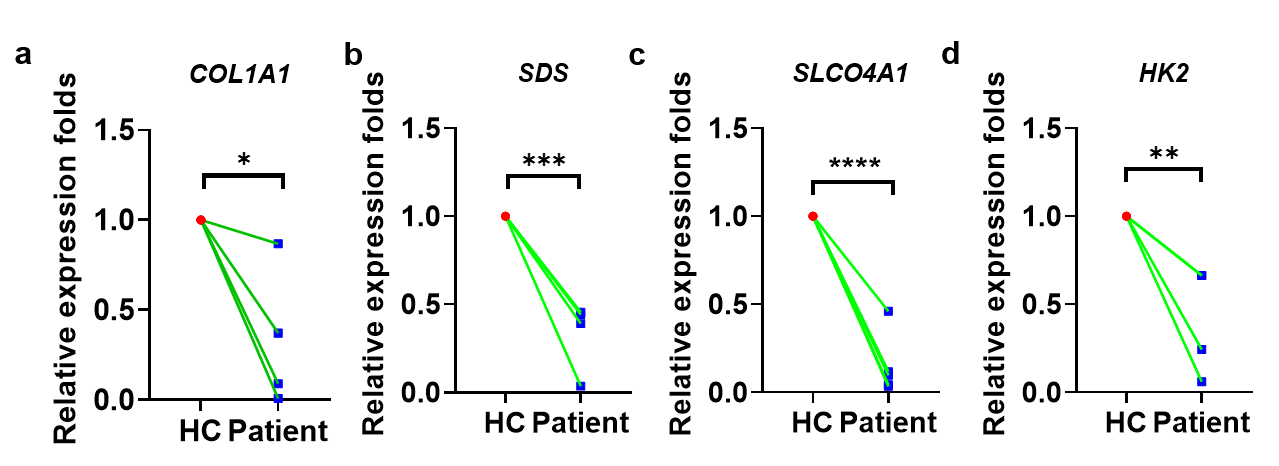


Supplemental Fig. S2. The changes of mRNA expression in recovered COVID-19 patients. Quantitative RT-PCR analysis of HK2, SLCO4A1, SDS, and COL1A1 in B cells from COVID-19 recovered patients and healthy controls (n=4) (a-d). Statistical evaluation was performed using the two-tailed Student’s *t*-test. Statistical significance is indicated using asterisks: **P* <0.05, ***P* < 0.01, ****P* < 0.001, and *****P* < 0.0001.
